# Health systems performance in managing tuberculosis: analysis of tuberculosis care cascades among high-burden and non-high-burden countries

**DOI:** 10.7189/jogh.09.010423

**Published:** 2019-06

**Authors:** Jungyeon Kim, Salmaan Keshavjee, Rifat Atun

**Affiliations:** 1 Department of Global Health and Population, Harvard T.H. Chan School of Public Health, Harvard University, Boston, Massachusetts A, USA; 2 Department of Global Health and Social Medicine, Harvard Medical School, Harvard University, Boston, Massachusetts, USA

## Abstract

**Background:**

Tuberculosis (TB) is a major global health burden, which has been inadequately addressed. This study aims to analyze different patterns and gaps of care along the care cascade across countries and to develop a model to examine the relationship between performance of tuberculosis programmes in high and low burden countries along the tuberculosis care cascade and tuberculosis disease burden.

**Methods:**

We used the World Health Organization’s Global TB Database for the year 2016 to construct tuberculosis care cascade consisting of four steps: incidence, diagnosed, treatment started and treatment completed. Based on the constructed care cascades, we analyzed the relationship between health system performance indicators and tuberculosis cascades performance: diagnosed rate, treatment started rate, and treatment completed rate.

**Results:**

There are wide differences in access to diagnosis and treatment between high-burden countries and non-high-burden countries. The largest gap was found between incidence and diagnosed rate, with 65% of diagnosed rate for high burden countries and 80% of diagnosed rate for non-high burden countries. We found variations in care performance among high-burden countries. We found a negative relationship between the population health indicators related to the mortality rate and TB care cascade performance. There was a positive relationship between immunization coverage rate and antenatal care indicators and TB care cascade performance.

**Conclusions:**

Well-functioning tuberculosis care cascades and effective health systems are important for the successful management of tuberculosis. While improving screening performance is essential for tuberculosis control especially for high-burden countries, resource should be allocated to improve health system performance, which is weak in high-burden countries. Performance of TB programmes across care cascade could be used as a useful tracer to measure performance of health systems.

Tuberculosis has been largely curable since 1948, yet it killed an estimated 1.8 million in 2015 [1-3]. Since 2010, the global tuberculosis incidence has declined at an unimpressive rate of around 1.5%-1.65% each year [1-3]. In 2015, there were an estimated 10.4 million new cases of tuberculosis [3], and of these, 4.3 million cases, around 40%, were either not diagnosed or were not notified to health authorities [3]. Tuberculosis continues to be the biggest killer of people living with HIV, and afflicts an estimated one million children, only one third of whom receive any diagnosis or care [3].

The End TB Strategy, adopted by the World Health Assembly in 2014, aims to reduce the incidence of tuberculosis by 80% and deaths by 90% by 2030 compared to the levels observed in 2015 [4]. Since 2000, coinciding with the launch of the United Nations’ Millennium Development Goals (MDGs), the development assistance for tuberculosis rose from US$ 150 million in 2000 to US$ 1.5 billion in 2016 [5], contributing to the expansion and improvement of tuberculosis control programs globally [6,7]. However, in 2015 the funding for tuberculosis from international sources was lower than that in 2010, due to reduced donor funding that followed the global economic downturn in 2009 [5,7]. The ongoing economic malaise experienced by donor countries, and the transition from MDGs to Sustainable Development Goals (SDGs), with many priority areas and targets beyond the MDGs, means increased donor funding for tuberculosis is unlikely [6,8]. Hence, available resources have to be used more effectively and efficiently to improve tuberculosis diagnosis, treatment, and care delivery.

Conditions such as diabetes, tuberculosis or HIV have been used as tracers to examine health system performance [9,10]. An analysis of the tuberculosis care cascade could be used to examine health system performance in relation to management of tuberculosis [11-13]. Earlier studies have documented the association between performance of national tuberculosis programs and tuberculosis disease burden [14-21]. These studies have shown that timely diagnosis and treatment resulted in better outcomes [14,16,19,22]. High performance in relation to case detection helped to lower tuberculosis incidence, prevalence, and mortality levels [14]. Conversely, under-diagnosis of tuberculosis and barriers to treatment access experienced in weak health systems undermined effective management of tuberculosis and adversely affected outcomes [1,19,21]. For example, in Ethiopia, delayed diagnosis and treatment of tuberculosis patients led to poor treatment outcomes [16]. Effective treatment is key to reducing tuberculosis deaths. Case fatality rate for untreated tuberculosis is estimated to be 70% globally [23]. Treatment completion is critically important in achieving better treatment outcomes, and good ambulatory care delivery may help limit emergence of drug-resistant tuberculosis [24-26].

This study examines patterns of care along tuberculosis care cascade in countries with high-burden of disease (as defined by the World Health Organization (WHO): 20 high tuberculosis burden countries based on absolute number of incident cases and 10 high tuberculosis burden countries based the prevalence rate per 100 000 population) and low-burden of tuberculosis, to ascertain performance patterns of health systems in managing tuberculosis and to show the care gaps across countries. We hypothesized that high-burden tuberculosis countries underperform in each area of the care cascade compared to non-high-burden countries. We also hypothesized that performance of tuberculosis care cascade is correlated with health system performance.

## METHODS

### Data

We used the WHO Global TB database [27], which includes data reported by 217 countries on notification, treatment, mortality and financing for tuberculosis. The data also includes estimated prevalence rates for tuberculosis in each country. We analyzed data from countries which reported tuberculosis notification cases in 2015 and treatment cases in 2014. The difference in years arises because WHO collects and releases treatment success rates based on treatment cohorts from the preceding year.

Our study was based on data from 183 countries, where the total number of incidence cases in 2015 was more than zero and the total number of treated or cured cases of 2014 cohort was more than zero. We excluded 9 countries that had missing values or zero for estimated number of incident cases.

### Analysis

#### TB care cascade

Using data from WHO Global TB database in 2015 we constructed a tuberculosis care cascade consisting of four steps: incidence, diagnosed, treatment started and treatment completed. We used (i) “estimated number of incident cases (all forms)” for ‘incidence’, (ii) the sum of “total of new and relapse cases and cases with unknown previous tuberculosis treatment” for ‘diagnosed’, (iii) the sum of “outcomes for all new and relapse cases: cohort size” and “outcomes for previously treated patients: cohort size” for ‘treatment started’, and (iv) sum of “outcomes for all new and relapse cases: treatment success (cured or treatment completed)” and “outcomes for previously treated patients: treatment success (cured or treatment completed)” for ‘treatment completed’.

The number of incident tuberculosis cases of 2015, the first step of cascade, was expressed as 100%. Each of the following tuberculosis care cascade steps (diagnosed, treatment started and treatment completed) was divided by WHO’s estimate of the number of incident tuberculosis cases for 2015 and was presented as a percentage.

If a country either reported new and relapse cases and cases with unknown previous tuberculosis treatment history, or new and relapse cases and cases with unknown previous tuberculosis treatment history, we treated the missing values of either variable as zero and summed both variables to get the total figure for the step of diagnosis. Likewise, if a country either reported treatment success cases of new and relapse cases, or treatment success cases of previously treated patients, we treated missing values of either variable as zero and summed both variables to get the total figure for the step of treatment.

To analyze and compare variations in tuberculosis care cascade of high-burden countries, we first developed one care cascade combining data for the 30 high-burden countries. We then developed 30 care cascades for each of 30 high- burden countries; one overall average cascade that included the 183 countries worldwide; and one overall average cascade for the 153 non-high-burden countries. We calculated the proportion of diagnosed, treatment started and treatment completed as percentages of incidence in each country and constructed aggregated care cascades by averaging the proportion of diagnosed, treatment started and treatment completed for each country in each study group.

Based on the constructed tuberculosis care cascades, we then performed a non-parametric Wilcoxon-Mann-Whitney test to compare the differences in performance in tuberculosis care cascades among the 30 high-burden countries and the 153 non-high-burden countries. We used non-parametric statistics because the variables analyzed were not normally distributed.

We also analyzed the relationship between indicators routinely used to assess overall health system performance, namely population health indicators (infant mortality rate [28], maternal mortality ratio [29], and death rate for communicable diseases and maternal, prenatal and nutrition conditions [30]), immunization coverage rates (for BCG [31], Hepatitis B [32], DPT [33], measles [34], polio [35]), and antenatal care indicators (pregnant women receiving prenatal care [36], pregnant women receiving prenatal care of at least four visits [37] and births attended by skilled health staff [38]) to examine if tuberculosis management performance, as measured by the care cascade, could also be used as a tracer to examine health system performance [39,40].

## RESULTS

In 2015, 206 countries reported 6.2 million of new and relapse tuberculosis cases and 201 countries reported 0.2 millions of previously treated cases.

The 30 high burden countries accounted for 87% of the global tuberculosis incidence: Angola, Bangladesh, Brazil, China, Democratic People’s Republic of Korea, Democratic Republic of the Congo, Ethiopia, India, Indonesia, Kenya, Mozambique, Myanmar, Nigeria, Pakistan, Philippines, Russian Federation, South Africa, Thailand, United Republic of Tanzania, Viet Nam (based on the number of incident cases), Cambodia, Central African Republic, Congo, Lesotho, Liberia, Namibia, Papua New Guinea, Sierra Leone, Zambia, Zimbabwe (based on the prevalence rate).

Eleven countries – Antigua and Barbuda, Aruba, US Virgin Islands, Comoros, Bonaire, Saint Eustatius and Saba, Saint Kitts and Nevis, San Marino, Bahrain, Curacao, Turkmenistan and Qatar- didn’t report their total new and relapse cases and cases with unknown previous tuberculosis treatment history in 2015.

Five countries – Japan, Greenland, Saint Lucia, Canada, and Haiti – reported new and relapse cases and cases with unknown previous tuberculosis treatment history but not previously treated patients in 2015.

193 countries reported treatment cases of all new and relapse cases of 2014 cohort (cured or treatment completed) in 2015, and 182 countries reported the number of treatment success cases (cured or treatment completed) of previously treated patients (cured or treatment completed) of 2014 cohort in 2015.

There were seven countries where the total notified cases were higher than the estimated number of tuberculosis cases incident in 2015. These seven countries (estimated incidence/the total number of notified tuberculosis cases) were Azerbaijan (6800/7501), Denmark (340/357), Nauru (12/18), Republic of Korea (40 000/40 847), Russian Federation (11 500/130 904), Sao Tome and Principe (180/207), and The Former Yugoslav Republic of Macedonia (270/284).

**Figure 1:**
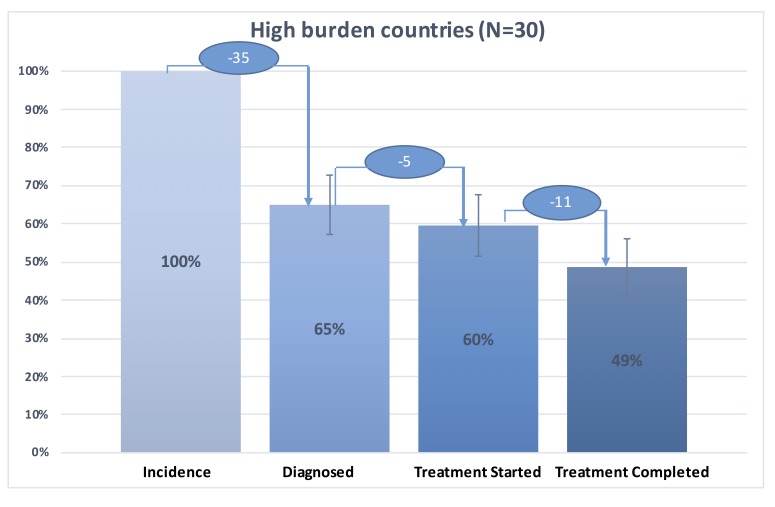
TB care cascade for 30 high-burden countries, 2015. Diagnosed: 100 × [(sum of total of new and relapse cases and cases with unknown previous tuberculosis treatment, 2015)/(estimated number of incident cases (all forms), 2015)]. Treatment Started: 100 × [(sum of outcomes for all new and relapse cases: cohort size and outcomes for previously treated patients: cohort size, 2015)/(estimated number of incident cases (all forms), 2015)]. Treatment Completed: 100 × [(sum of outcomes for all new and relapse cases: treatment success (cured or treatment completed) and outcomes for previously treated patients: treatment success (cured or treatment completed)/ (estimated number of incident cases (all forms), 2015)].

**Figure 2:**
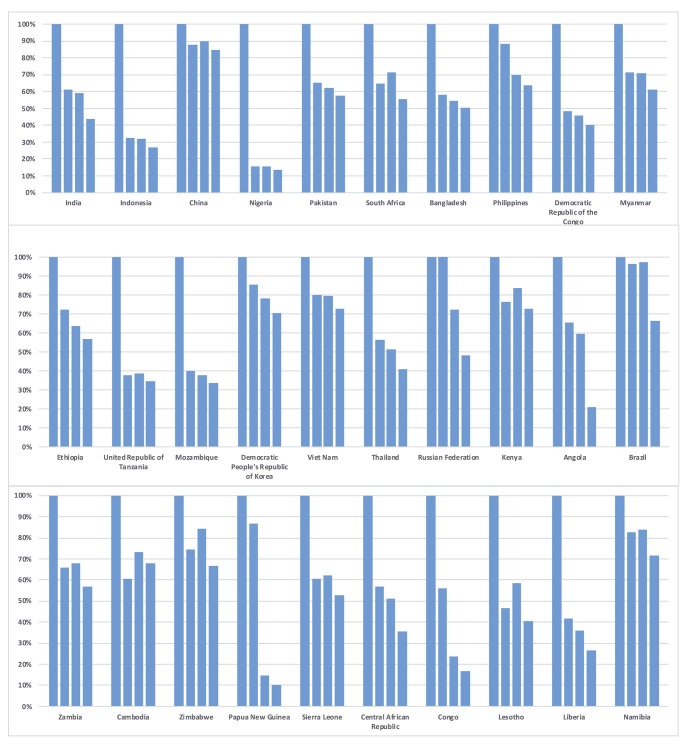
TB care cascade for each of 30 high burden countries, 2015. Steps correspond to, in order, Incidence, Diagnosed, Treatment Started, and Treatment Completed.

**Figure 3:**
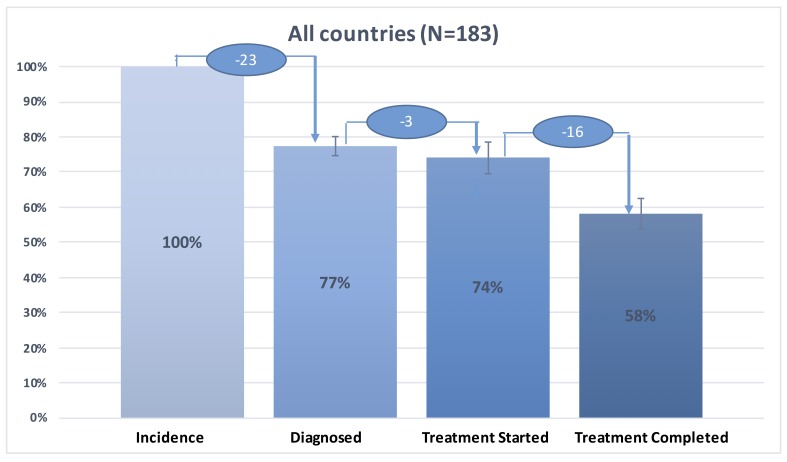
TB care cascade for all the 30 high burden and 153 non-high burden countries, 2015.

**Figure 4:**
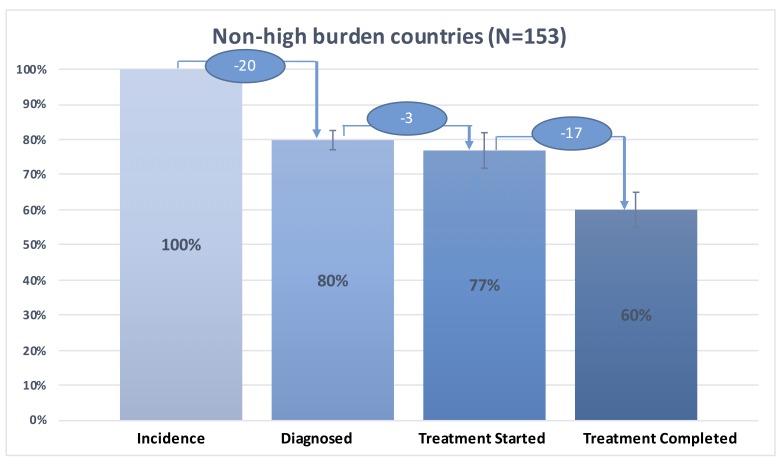
TB care cascade for 153 non-high burden countries, 2015.

We present in **Figure 1** the tuberculosis care cascade combining data for the 30 high-burden countries, in **Figure 2** the tuberculosis care cascades for each of 30 high-burden countries, in Figure 3, one overall average cascade that included the 183 countries worldwide, and in **Figure 4** one overall average cascade for the 153 non-high-burden countries.

There was a statistically significant difference in the diagnosis rate, treatment start rate, and treatment completion rate between the 30 high-burden countries and the 153 non-high-burden countries at the alpha level of *P* ≤ 0.05 (**Table 1**). The 30 high-burden countries had statistically significant lower rank in diagnosed rate and treatment start rate compared to 153 non-high burden countries.

**Table 1:** Wilcoxon-Mann-Whitney test for care cascade and TB burden between high burden countries and non-high burden countries

	N	Test statistics	*P*-value
Diagnosed	183	3.849	<0.001
Treatment Started	183	3.374	<0.001
Treatment Completed	183	2.167	0.030

*The number of treated patients was based on WHO 2014 data.

In the 30 high-burden countries, patients with tuberculosis were less likely to be diagnosed and treated compared to those who lived in non-high-burden countries (**Figure 1**, **Figure 3** and **Figure 4**). **Figure 1** illustrates the tuberculosis care cascade of average 30 high burden countries with 95% confidence interval. In the 30 high-burden countries, on average 65% (95% CI = 57%-73%) of tuberculosis population was diagnosed and approximately 49% (95% confidence interval CI: 41% -56%) out of tuberculosis population completed treatment. The 30 high-burden countries showed the biggest gap between tuberculosis incidence and individuals diagnosed with tuberculosis, compared to other countries.

**Figure 2** presents the tuberculosis care cascades for each of the 30 high-burden countries. Approximately 77% (23 countries of the 30 high-burden countries) underperform in case detection, with diagnosis rate less than 77.38%, which was the average diagnosis rate for the 183 countries. Nigeria showed the largest gap between new cases of tuberculosis and people who had access to diagnosis and treatment. Only 15% (90,584) of Nigerian population with presumptive tuberculosis (n = 586 000) was diagnosed, and just 13% (n = 78 883) of this group completed treatment. Conversely, Papua New Guinea showed comparatively high diagnosis rate (87%; 28 696 of 33 000), but showed the largest drop from diagnosis to treatment care step with the lowest treatment start rate (15%: 4805 of 33 000) and the lowest treatment completion rate (10%; 3293 of 33 000) among 30 high-burden countries. Indonesia, Nigeria, United Republic of Tanzania, Mozambique showed diagnosis rates lower than 40%. Even though China was ranked third among 30 high-burden countries in terms of the number of tuberculosis incident cases, it showed diagnosis, treatment start, and treatment completion rates higher than 85%. Among six countries that accounted for 60% of tuberculosis incidence in 2015, five countries except China, India, Indonesia, Nigeria, Pakistan, South Africa showed low screening performance with large gaps between tuberculosis prevalence and those diagnosed.

**Figure 3** presents aggregated average tuberculosis care cascade across 183 countries with 95% confidence interval. We estimated that 78% (95% CI = 75%-80%) was diagnosed, 74% (95% CI = 70%-79%) started treatment, and 58% (95% CI = 54%-63%) of tuberculosis population successfully completed treatment globally (**Figure 3**). 23% of the tuberculosis population was not diagnosed, 3% of diagnosed patients did not receive treatment, and 16% of patients who started treatment were lost to follow-up during the course of treatment (**Figure 3**).

**Figure 4** shows aggregated average tuberculosis care cascade for the 153 non-high burden countries. We estimated that 80% (95% CI = 77%-83%) of the cases was diagnosed, 77% (95% CI = 72%-82%) started treatment, and 60% (95% CI = 55%-65%) successfully completed treatment. The biggest difference between the tuberculosis care cascade for high-burden countries and that for non-high-burden countries was in the step of diagnosis. While 35% of tuberculosis population was undiagnosed in high-burden countries, around 20% of tuberculosis population was undiagnosed in the non-high-burden countries (**Figure 1** and **Figure4**). There was higher rate of treatment dropout rate (17%) in the non-high-burden countries compared to that in high-burden countries (11%) (**Figure 1** and **Figure 4**).

### Relationship between TB care cascade performance and health system performance indicators

We found an overall negative relationship between population health indicators (namely, infant mortality rate, maternal mortality ratio, the cause of death by communicable disease, and maternal, prenatal and nutrition conditions) typically used to measure health system performance and TB care cascade performance (**Table 2**) (ie, strong performance of TB care cascade was correlated with better population health outcomes, as indicated by lower mortality rates). The correlation coefficients ranged from -0.6 to -0.4 between performances of TB care cascade steps (diagnosed, treatment started and treatment completed) and the selected population health indicators (**Table 2**). The relationship between population health indicators and TB care cascade performance was biggest in the care cascade step of diagnosed with -0.6 of correlation coefficient (**Table 2**).

**Table 2:** Correlation between TB care cascade performance and health systems indicators in 2015

			TB care cascade performance		
**Health systems indicators**		**N**	**Diagnosed**	**Treatment started**	**Treatment completed**
Population	Infant mortality rate	175	-0.6	-0.5	-0.4
Health	Maternal mortality ratio	169	-0.6	-0.5	-0.4
	Cause of death, by communicable diseases and maternal, prenatal and nutrition conditions (% of total)	168	-0.6	-0.5	-0.4
Immunization	DPT	174	0.3	0.3	0.3
Coverage	Measles	174	0.4	0.4	0.4
Rate	Hepatitis B	166	0.3	0.3	0.3
	Holio	174	0.4	0.3	0.3
	HIB3	171	0.3	0.3	0.3
	BCG	149	0.2	0.2	0.2
Antenatal	Pregnant women receiving prenatal care (%)	38	0.3	0.3	0.3
Care	Pregnant women receiving prenatal care of at least four visits (% of pregnant women)	35	0.3	0.2	0.2
	Births attended by skilled health staff (% of total)	90	0.5	0.4	0.3

DPT – diphtheria, pertussis, tetanus, BCG – Bacillus Calmette-Guerin, HIB3 – Haemophilus influenza type B

We found a positive relationship between immunization coverage rate and antenatal care coverage and TB care cascade performance (ie, strong performance of TB care cascade was positively correlated better immunization rate and antenatal care coverage) (**Table 2**). The correlation coefficients ranged from 0.2 to 0.4 for the relationship between immunization coverage rate indicators and TB care cascade performance and those ranged from 0.2 to 0.5 for the relationship between antenatal care indicators and TB care cascade performance (**Table 2**).

## DISCUSSION

In this study we constructed tuberculosis care cascades to examine patterns of tuberculosis care between high-burden tuberculosis countries and non-high-burden countries. The results of this study supported our hypothesis that high-burden countries underperform across the tuberculosis care cascade compared to non-high-burden countries, revealing wide differences in diagnosis rate, treatment start rate, and treatment completion rate across countries. The biggest difference in the care cascade performance between high-burden countries and non-high-burden countries occurred in the diagnosis step, showing that a substantial number of presumptive tuberculosis patients remained undiagnosed particularly in high-burden countries.

There were distinct features of the tuberculosis care cascade among the 30 high-burden countries. Nigeria had the lowest diagnosis rate (less than 20%), and 85% of the new cases of tuberculosis remained undiagnosed and did not receive proper care. On the other hand, regardless of good performance in diagnosis, tuberculosis patients in Papua New Guinea and Angola did not receive treatment for their disease.

We found an overall negative relationship between population health indicators, such that countries with poorly performing care cascades also had worse health outcomes and coverage of key health services (such as immunization and antenatal care). Our findings are in line with earlier studies that suggested successful tuberculosis control depends on well-functioning health systems and effective health services provision [14-21]. Especially for the high-burden countries, the biggest challenge to tuberculosis control was in diagnosis. Performance in relation to diagnosis was identified as the care cascade step with strongest relationship with population health indicators. The results indicate potential use of tuberculosis care cascades as a tracer to assess overall health system performance – a useful tracer, as tuberculosis treatment lasts several months and provides an indication of how well a health system is providing continuity of care.

Low health system performance will impede the achievement of the tuberculosis targets set in the End TB Strategy and in the SDGs. Considering the current global funding constraints in Development Assistance for Health, resources should be allocated to improve health systems in high-burden tuberculosis countries. While low screening performance was a consistent feature of high-burden countries, performance patterns varied, and this variation, which indicates where health system weaknesses exist in the care cascade, could be used to inform targeted health system interventions. Our study indicated key areas that each of 30 high-burden countries should focus their activities to improve TB program performance. Based on the biggest gap along the care cascades from our **Figure 2**, we may categorize countries into three groups depending on the area they should prioritize [41]. In most of the high-burden countries (such as India, Indonesia, China, Nigeria, Pakistan, South Africa, Bangladesh, Democratic Republic of Congo, Myanmar, Ethiopia, United Republic of Tanzania, Mozambique, Democratic People’s Republic of Korea, Viet Nam, Thailand, Kenya, Zambia, Cambodia, Zimbabwe, Sierra Leone, Central African Republic, Congo, Lesotho, Liberia, Namibia), resources for tuberculosis control should be prioritized to improve screening performance through increased screening and active case finding [42]. For the Philippines, Russian Federation and Papua New Guinea where the biggest gaps exist between ‘diagnosis’ and ‘treatment started’, resources should be prioritized to expand treatment service provision to put all the people who are diagnosed with tuberculosis on treatment. In both Angola and Brazil, the health systems should be strengthened to reduce ‘treatment loss to follow-up’ [42].

Although our study has demonstrated and quantified the differential gap in tuberculosis care cascades across countries and how performance of care cascade correlates with health systems performance, our analysis has several limitations. First, we used the cross-sectional data of 2015 for estimated prevalence, mortality and diagnosed cases but used the 2014 cohort data for treatment start cases and for treatment completion cases to construct tuberculosis care cascade and models. This may produce a potential bias in treatment start performance and treatment completion performance, because, theoretically, there is a possibility of abrupt improvement or decline in treatment rates from 2014 to 2015 treatment cohort (although in practice this is highly unlikely). A longitudinal study to examine the trend differences in tuberculosis outcome and tuberculosis care performance would help understand better the relationship between tuberculosis burden and tuberculosis care performance.

Second, the estimated incidence of tuberculosis has an uncertainty. Our cascade was built using WHO’s estimated tuberculosis incidence figures. According to WHO, the estimate of tuberculosis incidence for 2015 is based on “case notification data combined with expert opinion about case detection gaps,” “results from tuberculosis prevalence surveys,” “notifications in high-income countries adjusted by a standard factor to account for under-reporting and under-diagnosis,” and “results from inventory/capture-recapture studies [43].” There were seven countries where the total notified cases were higher than the estimated number of tuberculosis incident in 2015. To improve our models, we used the upper bound of estimated number of tuberculosis incident cases for these countries to ascertain the effect of health system performance on tuberculosis disease burden. This approach may have resulted in underreporting the figures in the care cascades.

Despite these limitations, however, the study sheds new light on patterns of the management of tuberculosis across countries and has important policy implications. We constructed care cascades for each high-burden tuberculosis country that enabled us to examine differences in performance across countries in each step of the cascade and to identify areas for improvement.

Results of our analysis indicate that diagnosis performance was the most critical element of the cascade in reducing tuberculosis disease burden and improving outcome. Findings from our study suggest that while overall health system performance in high-burden countries needs to be improved, the step of improved diagnosis needs to be prioritized to increase overall performance along the care cascade and improve tuberculosis outcomes.

## References

[1] Yuen CM, Amanullah F, Dharmadhikari A, Nardell EA, Seddon JA, Vasilyeva I (2015). Turning off the tap: stopping tuberculosis transmission through active case-finding and prompt effective treatment.. Lancet.

[2] Ortblad KF, Lozano R, Murray CJ (2013). An alternative estimation of tuberculosis incidence from 1980 to 2010: methods from the Global Burden of Disease 2010.. Lancet.

[3] World Health Organization. Global tuberculosis report 20162016.

[4] World Health Organization. The end TB strategy2014.

[5] Institute for Health Metrics and Evaluation. Financing Global Health 2016. Available: http://www.healthdata.org/sites/default/files/files/policy_report/FGH/2017/IHME_FGH2016_Technical-Report.pdf. Accessed: 28 February 2019.

[6] World Health Organization. Health in 2015: from MDGs, Millennium Development Goals to SDGs. 2015.

[7] Dieleman JL, Schneider MT, Haakenstad A, Singh L, Sadat N, Birger M (2016). Development assistance for health: past trends, associations, and the future of international financial flows for health.. Lancet.

[8] Atun R, Knaul FM, Akachi Y, Frenk J (2012). Innovative financing for health: what is truly innovative?. Lancet.

[9] Travis P, Bennett S, Haines A, Pang T, Bhutta Z, Hyder AA (2004). Overcoming health-systems constraints to achieve the Millennium Development Goals.. Lancet.

[10] Atun R, Jaffar S, Nishtar S, Knaul FM, Barreto ML, Nyirenda M (2013). Improving responsiveness of health systems to non-communicable diseases.. Lancet.

[11] Papanicolas I, Smith P. Health system performance comparison: an agenda for policy, information and research: an agenda for policy, information and research: McGraw-Hill Education (UK); 2013.

[12] Linas BP, Wong AY, Freedberg KA, Horsburgh CR (2011). Priorities for screening and treatment of latent tuberculosis infection in the United States.. Am J Respir Crit Care Med.

[13] Pooran A, Booth H, Miller RF, Scott G, Badri M, Huggett JF (2010). Different screening strategies (single or dual) for the diagnosis of suspected latent tuberculosis: a cost effectiveness analysis.. BMC Pulm Med.

[14] Akachi Y, Zumla A, Atun R (2012). Investing in improved performance of national tuberculosis programs reduces the tuberculosis burden: analysis of 22 high-burden countries, 2002–2009.. J Infect Dis.

[15] Khan AJ, Khowaja S, Khan FS, Qazi F, Lotia I, Habib A (2012). Engaging the private sector to increase tuberculosis case detection: an impact evaluation study.. Lancet Infect Dis.

[16] Madebo T, Lindtjorn B (1999). Delay in Treatment of pulmonary tuberculosis: An analysis of symptom duration among Ethiopian patients.. MedGenMed.

[17] Pablos-Méndez A, Sterling TR, Frieden TR (1996). The relationship between delayed or incomplete treatment and all-cause mortality in patients with tuberculosis.. JAMA.

[18] Rajeswari R, Chandrasekaran V, Suhadev M, Sivasubramaniam S, Sudha G, Renu G (2002). Factors associated with patient and health system delays in the diagnosis of tuberculosis in South India.. Int J Tuberc Lung Dis.

[19] Storla DG, Yimer S, Bjune GA (2008). A systematic review of delay in the diagnosis and treatment of tuberculosis.. BMC Public Health.

[20] Yimer S, Bjune G, Alene G (2005). Diagnostic and treatment delay among pulmonary tuberculosis patients in Ethiopia: a cross sectional study.. BMC Infect Dis.

[21] Atun R, Weil DE, Eang MT, Mwakyusa D (2010). Health-system strengthening and tuberculosis control.. Lancet.

[22] Lawn SD, Afful B, Acheampong JW (1998). Pulmonary tuberculosis: diagnostic delay in Ghanaian adults.. Int J Tuberc Lung Dis.

[23] Tiemersma EW, van der Werf MJ, Borgdorff MW, Williams BG, Nagelkerke NJ (2011). Natural history of tuberculosis: duration and fatality of untreated pulmonary tuberculosis in HIV negative patients: a systematic review.. PLoS One.

[24] Orenstein EW, Basu S, Shah NS, Andrews JR, Friedland GH, Moll AP (2009). Treatment outcomes among patients with multidrug-resistant tuberculosis: systematic review and meta-analysis.. Lancet Infect Dis.

[25] World Health Organization. Treatment of tuberculosis: guidelines. Geneva: World Health Organization; 2010.

[26] Laserson KF, Thorpe L, Leimane V, Weyer K, Mitnick C, Riekstina V (2005). Speaking the same language: treatment outcome definitions for multidrug-resistant tuberculosis.. Int J Tuberc Lung Dis.

[27] World Health Organization. Global Health Observatory (GHO) data: Tuberculosis. 2017. Available: http://www.who.int/gho/en/. Accessed: 15 January 2018.

[28] UNICEF, WHO, Word Bank, Division UDP. Mortality rate, infant (per 1,000 live births). In: World Bank Group, editor. 2015. https://childmortality.org Accessed 15 January 2018.

[29] WHO. UNICEF, UNFPA, World Bank Group, Divisions UP. Maternal mortality ratio (modeled estimate, per 100,000 live births). In: World Bank Group, editor. 2015. https://data.worldbank.org/indicator/SH.STA.MMRT Accessed 15 January 2018.

[30] World Health Organization. Cause of death, by communicable disease and maternal, prenatal and nutrition conditions (% of total). In: World Bank Group, editor. 2015. Available: https://data.worldbank.org/indicator/SH.DTH.COMM.ZS?view=chart. Accessed 15 January 2018.

[31] WHO. UNICEF. Immunization, BCG (% of one-year-old children). In: World Bank Group, editor. 2015. Available: http://www.who.int/immunization/monitoring_surveillance/en/ Accessed: 15 January 2018.

[32] WHO. UNICEF. Immunization, HepB3 (% of one-year-old children). In: World Bank Group, editor. 2015. Available: http://www.who.int/immunization/monitoring_surveillance/en/ Accessed 15 January 2018.

[33] World Health Organization. UNICEF. Immunization, DPT (% of children ages 12-23 months). In: World Bank Group, editor. 2015. Available: http://www.who.int/immunization/monitoring_surveillance/en. Accessed: 15 January 2018.

[34] World Health Organization. UNICEF. Immunization, measles (% of children ages 12-23 months). In: World Bank Group, editor. 2015. http://www.who.int/immunization/monitoring_surveillance/en Accessed 15 January 2018.

[35] World Health Organization. UNICEF. Immunization, Pol3 (% of one-year-old children) In: World Bank Group, editor. 2015. Available: http://www.who.int/immunization/monitoring_surveillance/en/. Accessed: 15 January 2018.

[36] UNICEF. State of the World's Children, Childinfo, Demographic and Health Surveys. Pregnant women receiving prenatal care (%). In: World Bank Group, editor. 2015. Available: http://databank.worldbank.org/data/reports.aspx?source=health-nutrition-and-population-statistics. Accessed: 15 January 2018.

[37] UNICEF. State of the World's Children, Childinfo, Demographic and Health Surveys. Pregnant women receiving prenatal care of at least four visits (% of pregnant women). In: World Bank Group, editor. 2015. Available: http://databank.worldbank.org/data/reports.aspx?source=health-nutrition-and-population-statistics. Accessed: 15 January 2018.

[38] UNICEF. State of the World's Children, Childinfo, Demographic and Health Surveys. Births attended by skilled health staff (% of total). In: World Bank Group, editor. 2015. Available: https://data.worldbank.org/indicator/SH.STA.BRTC.ZS Accessed: 15 January 2018.

[39] Smith PC. Performance measurement for health system improvement: experiences, challenges and prospects. Cambridge: Cambridge University Press; 2009.

[40] The World Bank. World Development Indicators. In: The World Bank, editor. 2017. Available: http://databank.worldbank.org/data/reports.aspx?source=world-development-indicators&Type=TABLE&preview=on# Accessed 14 August 2017.

[41] Naidoo P, Theron G, Rangaka MX, Chihota VN, Vaughan L, Brey ZO (2017). The South African tuberculosis care cascade: estimated losses and methodological challenges.. J Infect Dis.

[42] Harries AD, Lin Y, Kumar AM, Satyanarayana S, Takarinda KC, Dlodlo RA (2018). What can national TB control programmes in low-and middle-income countries do to end tuberculosis by 2030?. F1000Research.

[43] Glaziou P, Sismanidis C, Zignol M, Floyd K. Methods used by WHO to estimate the global burden of TB disease. Global TB Programme, World Health Organization, Geneva, Switzerland, 2016 Available: https://www.who.int/tb/publications/global_report/gtbr2016_online_technical_appendix_global_disease_burden_estimation.pdf?ua=1. Accessed: 1 August 2017.

